# Systematic review of mapping-based teaching strategies in pediatric nursing education: effects on learning outcomes among nurses and nursing students

**DOI:** 10.1186/s12909-026-08674-1

**Published:** 2026-02-17

**Authors:** Murad A. Sawalha, Mahmoud H. Alrabab’a, Rami A. Elshatarat, Saleh Mahmoud Mayyas, Amal A. Murad, Raghad Abdelkader, Khaldoon Aied Alnawafleh, Amna Nagaty Aboelmagd, Zyad T. Saleh, Khaled M. Al-Sayaghi

**Affiliations:** 1 Department of maternal, child and family health nursing, Faculty of Nursing, The Hashemite University, Zarqa, Jordan; 2 Prince Al‑Hussein Bin Abdullah II Academy for Civil Protection, Al‑Balqa Applied University, Salt, Jordan; 3 Department of Medical and Surgical Nursing, College of Nursing, Taibah University, Madinah, Saudi Arabia; 4 Department of Nursing Education, Madinah Cardiac Center, Ministry of Health, Madinah, Saudi Arabia; 5 Maternity and childhood nursing department, College of Nursing, Taibah University, Madinah, Saudi Arabia; 6 Maternal and child Heath Nursong Department, Faculty of Nursing, Applied Science Private University, Amman, Jordan; 7 Medical- Surgical Nursing Department, Faculty of Nursing, University of Tabuk, Tabuk, Saudi Arabia; 8 Department of Pediatric Nursing, Faculty of Nursing, Minia University, Minia, Egypt; 9 Department of Pediatric Nursing, Al-Ghad College for Applied Medical Sciences, Madinah, Saudi Arabia; 10 Department of Clinical Nursing, School of Nursing, The University of Jordan, Amman, Jordan; 11 Nursing Division, Faculty of Medicine and Health Sciences, Sana’a University, Sana’a, Yemen

**Keywords:** Concept mapping, Mind mapping, Pediatric nursing education, Learning outcomes, Nursing students, Pediatric nurses, Educational strategies

## Abstract

**Background:**

Concept mapping and mind mapping are increasingly applied in nursing education to strengthen critical thinking, knowledge integration, and clinical performance. In pediatric nursing, these strategies hold promise for enhancing both professional competence among practicing nurses and academic outcomes for nursing students, ultimately improving the quality of care for children and their families.

**Objective:**

This systematic review aimed to evaluate the effectiveness of concept mapping and mind mapping as educational interventions for pediatric nurses and nursing students, with a focus on their impact on learning outcomes and professional practice.

**Methods:**

A systematic literature review was conducted in accordance with PRISMA 2020 guidelines. Searches were performed across six major databases, yielding 988 initial records. Following screening and critical appraisal using the Joanna Briggs Institute (JBI) checklists, 12 studies met the eligibility criteria, including 9 studies involving nursing students and 3 involving pediatric nurses. Eligible studies were published between 2010 and 2025, employed experimental or quasi-experimental designs, and assessed outcomes such as knowledge acquisition, clinical performance, critical thinking, and self-efficacy. Data were extracted using a standardized form, and a narrative synthesis was applied due to heterogeneity in interventions and outcomes.

**Results:**

Evidence consistently indicated that concept mapping and mind mapping were effective educational strategies for both pediatric nurses and nursing students. Among pediatric nurses, interventions improved knowledge of congenital heart disease, infection control, and peripherally inserted central catheter (PICC) care, while also enhancing clinical performance and training satisfaction. Among nursing students, the interventions were associated with improved critical thinking, self-efficacy, problem-solving, academic achievement, cardiopulmonary resuscitation (CPR) skill acquisition, and overall satisfaction with learning.

**Conclusion:**

Concept mapping and mind mapping are effective educational methods for strengthening pediatric nursing education and practice. They enhance knowledge, clinical reasoning, self-efficacy, and satisfaction among both pediatric nurses and nursing students. Integration of these strategies into curricula and professional development programs is recommended. Future research should focus on standardized interventions, long-term evaluations, and direct measures of patient-centered outcomes to establish the sustained impact of these strategies on pediatric care.

## Introduction

The advancement of nursing education requires innovative pedagogical strategies that go beyond traditional lecture-based methods to promote deeper learning, critical thinking, and clinical reasoning. In pediatric nursing education, where learners must rapidly integrate complex developmental, physiological, and psychosocial factors to make safe clinical decisions, concept mapping and mind mapping have gained increasing attention as structured educational strategies [1, 2]. For example, Ayed et al. [3] conducted a quasi-experimental study in a neonatal intensive care unit, demonstrating that pediatric nurses who participated in a mind mapping training program significantly improved their infection control practices and clinical performance [3]. Similarly, Aein and Aliakbari [4] reported that nursing students in an experimental pediatric course who used concept mapping achieved higher critical thinking scores on the California Critical Thinking Skills Test compared with those in traditional lecture-based instruction [4]. Abdelrahman et al. [5] also found that concept mapping significantly enhanced nursing students’ knowledge and satisfaction when learning about congenital heart disease in pediatric settings [5]. These mapping-based approaches enable pediatric nurses and nursing students to systematically organize, integrate, and visually represent patient information and care processes, thereby strengthening critical thinking, clinical reasoning, and safe decision-making in pediatric care [6–9].

Concept mapping, first developed by Joseph Novak in the 1970s, is firmly rooted in constructivist learning theory and is designed to promote meaningful learning through the visual organization of knowledge [10, 11]. It involves the graphical representation of concepts arranged hierarchically and connected by linking words or phrases, allowing learners to explicitly identify relationships between ideas and integrate new information with existing cognitive structures [12, 13]. Within nursing education, this approach has been widely adopted to enhance understanding, critical thinking, and clinical reasoning by making complex knowledge structures visible and logically organized [1, 14–16]. In pediatric nursing education, concept mapping is particularly valuable because it supports structured clinical reasoning and systematic problem-solving in situations that require the integration of multifaceted physiological, developmental, and psychosocial information. By visually linking patient assessment data with nursing diagnoses, interventions, and expected outcomes, learners are better able to comprehend clinical priorities and anticipate patient responses [17, 18]. For example, concept mapping has been used to organize the pathophysiology, clinical manifestations, nursing diagnoses, and management strategies associated with congenital heart disease, to structure assessment findings and evidence-based interventions for neonatal sepsis, to integrate medication calculations with safety precautions and monitoring requirements in pediatric drug administration, and to design comprehensive nursing care plans for children experiencing respiratory distress across acute and recovery phases [19–22]. Through these applications, concept mapping fosters analytical thinking, enhances the accuracy of clinical judgment, and strengthens the integration of theoretical knowledge with clinical practice in pediatric nursing contexts [1, 14, 15].

Mind mapping is conceptually related to concept mapping but differs in both structure and cognitive emphasis. Rather than relying on hierarchical, proposition-based relationships, mind mapping adopts a non-linear, radial format in which a central idea is placed at the core and expanded outward through branches composed of keywords, symbols, colors, and images. This structure is designed to mirror associative thinking processes, thereby stimulating creativity, enhancing memory retention, and supporting rapid recall of information [12, 18, 23–25]. By encouraging learners to make flexible connections between ideas, mind mapping promotes a holistic approach to learning that is particularly effective for brainstorming, content revision, and synthesizing large volumes of information [12, 26]. Within pediatric nursing education, mind mapping has demonstrated particular value in fostering learner engagement, accommodating visual learning preferences, and promoting active participation in both classroom and clinical environments. Its application enables students and nurses to summarize and recall complex content efficiently, such as growth and developmental milestones across pediatric age groups, infection prevention and control measures in neonatal and pediatric intensive care units, and differential diagnoses with corresponding nursing priorities for common pediatric emergencies [12, 27, 28]. Mind mapping is also frequently used to facilitate collaborative learning during pediatric case discussions and simulation debriefings, where shared visual representations support collective reasoning and reflection. Through these applications, mind mapping enhances knowledge recall, increases learner motivation, and strengthens interactive learning experiences, thereby complementing more structured approaches such as concept mapping in pediatric nursing education [26, 29].

Recent reviews and meta-analyses have demonstrated the educational benefits of concept mapping in nursing education broadly. For example, Faraji et al. [1] synthesized findings from 44 studies and reported significant improvements in students’ and nurses’ educational performance [1], while Yue et al. [2] confirmed that concept mapping enhances critical thinking skills and dispositions compared with conventional teaching [2]. Despite these findings, research has pointed to the need for further investigation in specialized areas such as pediatric nursing, where unique challenges and learning needs exist [30].

In pediatric nursing, both practicing nurses and nursing students encounter distinct learning demands, ranging from understanding developmental milestones to managing high-acuity conditions in neonatal and pediatric intensive care units [5, 23, 31]. Educational strategies such as concept mapping and mind mapping may support these learners by bridging theoretical knowledge with clinical application, particularly in high-risk and fast-paced care environments. Empirical studies conducted in Egypt, Iran, Türkiye, and Thailand have reported improvements in pediatric nurses’ knowledge, infection control practices, and clinical performance, as well as nursing students’ critical thinking, self-efficacy, and academic achievement following mapping-based educational interventions [4–8, 32–34].

### Study objectives

Accordingly, this systematic review aims to: (1) evaluate the effectiveness of concept mapping and mind mapping on knowledge acquisition and clinical skill development among pediatric nurses and nursing students; (2) examine the impact of these strategies on critical thinking, clinical reasoning, and self-efficacy in pediatric nursing education; (3) compare the educational outcomes of mapping-based strategies with traditional teaching methods in pediatric clinical and academic settings; and (4) identify gaps in the existing literature to inform future experimental and quasi-experimental research in pediatric nursing education.

By synthesizing evidence from experimental, quasi-experimental, and qualitative studies, this review seeks to provide a comprehensive understanding of how concept mapping and mind mapping influence learning outcomes in pediatric nursing. The findings are intended to inform educators, curriculum developers, and policymakers in advancing evidence-based pedagogical practices that enhance professional competence and ultimately improve pediatric patient care [30, 35–37].

## Background and overview

Concept mapping has become a widely recognized pedagogical strategy in nursing education, with numerous studies demonstrating its effectiveness in enhancing learning outcomes, critical thinking, and clinical competence. Systematic reviews and meta-analyses have confirmed its impact on higher-order cognitive skills, learner satisfaction, and overall academic performance [1, 2]. Longitudinal and experimental studies further indicate that concept mapping contributes to sustained improvements in critical thinking and clinical decision-making among nursing students [15, 35, 38, 39].

In pediatric nursing specifically, concept mapping has demonstrated significant benefits for both practicing nurses and students. Abdelrahman et al. [5] reported improved knowledge and satisfaction among pediatric nurses learning about congenital heart diseases [5], while Ayed et al. [3] and Hegazy et al. [23] found that mind mapping enhanced infection control and central line care practices in neonatal intensive care units [3, 23]. For nursing students, concept mapping has been shown to foster academic achievement, critical thinking, and self-efficacy in pediatric courses and clinical practice [4, 6–8, 36]. Additionally, studies comparing mapping with conventional or case-based learning highlight its superiority in teaching cardiopulmonary resuscitation skills, medication administration, and diagnostic reasoning [26, 40].

Evidence from broader nursing contexts further reinforces the adaptability and value of mapping-based strategies. Concept mapping has been associated with improvements in practical skill performance, problem-solving, and clinical reasoning across a range of nursing specialties [27, 32, 41, 42]. Technology-enhanced mapping approaches, including mobile-based learning and virtual platforms, have also been shown to increase self-efficacy and knowledge acquisition, suggesting new opportunities for integration into modern nursing curricula [43, 44]. Meanwhile, qualitative studies highlight that students perceive concept maps as valuable tools for understanding complex subjects like anatomy and physiology, although practical challenges related to contextualization remain [45].

Together, this growing body of evidence demonstrates that concept mapping and mind mapping not only support cognitive development and knowledge retention but also enhance confidence, problem-solving, and professional competence [2, 15]. Within pediatric nursing, these strategies offer particular promise for bridging theory and practice, preparing students for complex clinical scenarios, and supporting nurses in delivering safe, evidence-based care to children and families [30, 34]. However, despite promising outcomes, variations in study design, sample characteristics, and cultural contexts underscore the need for systematic synthesis to provide more robust and generalizable conclusions. This review addresses that gap by critically evaluating the role of mapping-based teaching strategies in pediatric nursing education among both students and practicing nurses.

## Methods

### Study design

This review followed a structured literature review design to gather, analyze, and synthesize research evidence on the effectiveness of mapping-based teaching strategies in pediatric nursing education. The primary aim was to explore how concept mapping contributes to knowledge acquisition and learning outcomes among two key populations: pediatric nurses working in clinical practice and nursing students engaged in pediatric or child health courses. By examining both professional and pre-licensure educational contexts, the review provided a broad understanding of the role of concept mapping in enhancing pediatric nursing education.

### Eligibility criteria

Eligibility criteria were established a priori to ensure methodological rigor, transparency, and reproducibility. Studies were eligible for inclusion if they satisfied all of the following conditions: (1) publication in peer-reviewed journals between 2010 and 2025; (2) availability in full text and written in English; (3) focus on pediatric nurses working in clinical settings or nursing students enrolled in pediatric or child health nursing courses; (4) use of experimental or quasi-experimental study designs; (5) implementation of concept mapping and/or mind mapping as the primary educational intervention; and (6) reporting of quantitative outcomes related to knowledge acquisition, critical thinking, clinical reasoning, self-efficacy, clinical performance, learning achievement, or adherence-related clinical behaviors relevant to pediatric nursing practice, such as medication administration accuracy or compliance with clinical protocols.

To ensure specificity and methodological quality, only studies employing clearly defined, structured, and empirically supported measurement instruments were included. Eligible tools comprised standardized critical thinking assessments (e.g., California critical thinking skills test, Watson–Glaser critical thinking appraisal), validated self-efficacy scales (e.g., nursing caring self-efficacy scale, self-efficacy scale for medication administration in children), structured academic and achievement tests, nursing process and concept map scoring rubrics, clinical skills checklists (e.g., cardiopulmonary resuscitation skills checklists), and observational tools evaluating clinical performance and adherence to practice standards, including infection control and medication administration procedures. Studies assessing adherence-related outcomes were included only when such outcomes were measured using structured questionnaires, observational checklists, or validated performance assessment tools. When available, information on the psychometric properties of the measurement instruments—such as evidence of content or construct validity and reliability indices (e.g., Cronbach’s alpha, inter-rater reliability)—was extracted and considered to support the robustness of the evidence.

Studies were excluded if they were review articles, conference abstracts, editorials, dissertations, or employed non-experimental designs, including descriptive, survey-based, or qualitative-only approaches. Additionally, studies involving non-pediatric populations (e.g., adult nurses, physicians, or allied health professionals) or those lacking objective, quantifiable educational or clinical performance outcomes were excluded.

For analytical purposes, outcomes from the included studies were synthesized narratively. Findings were organized according to population group (pediatric nurses or nursing students), type of mapping strategy (concept mapping or mind mapping), and outcome domain (knowledge, critical thinking, self-efficacy, clinical performance, or adherence-related behaviors). This approach facilitated systematic comparison of intervention effects while appropriately addressing heterogeneity in study designs, instruments, and educational contexts.

### Search strategy

**Fig. 1:**
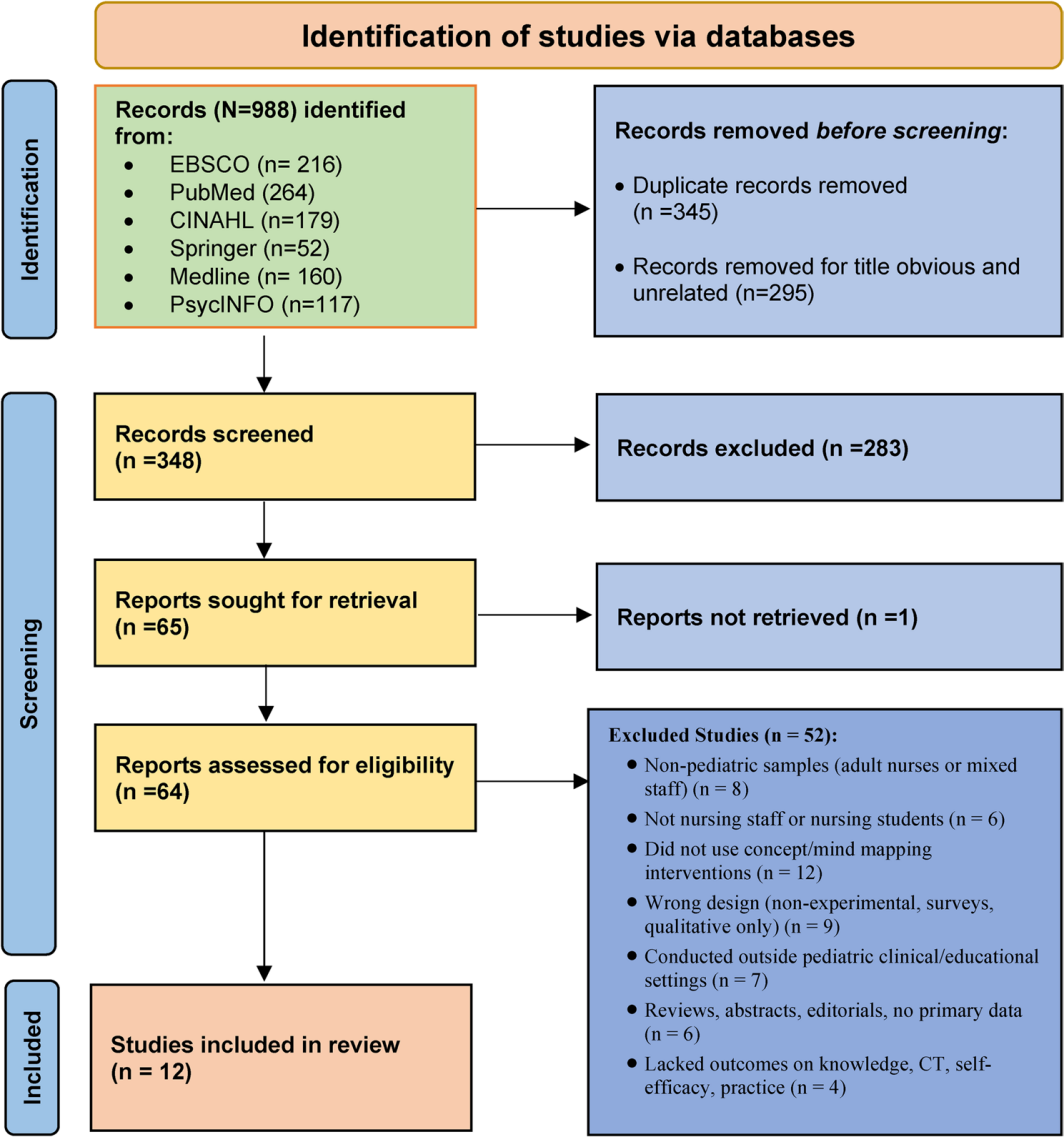
PRISMA flow chart of database search

A comprehensive and systematic search was undertaken across six major international electronic databases: EBSCO (*n* = 216), PubMed (*n* = 264), CINAHL (*n* = 179), Springer (*n* = 52), Medline (*n* = 160), and PsycINFO (*n* = 117), yielding a total of 988 records (Fig. 1). These databases were purposefully selected because of their broad and complementary coverage of nursing, medical, educational, and psychological research, thereby maximizing the likelihood of capturing all relevant empirical studies in pediatric nursing education.

The search strategy was explicitly structured according to the PICOS framework (Population, Intervention, Comparison, Outcomes, and Study design) to ensure conceptual clarity, methodological rigor, and reproducibility. Within this framework, the population comprised pediatric nurses and nursing students working or studying in pediatric clinical or educational settings; the intervention included concept mapping and/or mind mapping–based educational strategies; the comparison, where applicable, involved traditional teaching approaches or alternative instructional methods; the outcomes focused on knowledge acquisition, critical thinking, clinical reasoning, self-efficacy, and clinical performance; and eligible study designs were limited to experimental and quasi-experimental studies.

To facilitate exact replication, the search process was implemented in a sequential and standardized manner. First, controlled vocabulary terms (such as Medical Subject Headings in PubMed) relevant to concept mapping, mind mapping, pediatric nursing, and learning outcomes were identified. Second, equivalent free-text keywords and synonyms were developed to capture studies not indexed under controlled terms. Third, these terms were systematically combined using Boolean operators, whereby concept mapping–related terms (“concept mapping,” “concept map,” or “mind mapping”) were linked with pediatric nursing–related terms (“pediatric nursing,” “child health nursing,” “pediatric nurses,” or “nursing students”), and further combined with outcome-related terms (“knowledge acquisition,” “learning outcomes,” “clinical performance,” or “critical thinking”).

Subsequently, database-specific filters were applied to restrict results to studies published between 2010 and 2025, written in English, and aligned with the targeted study designs. The search syntax was carefully adapted to meet the indexing and search requirements of each database while preserving semantic equivalence across platforms. To enhance completeness, the reference lists of all included studies were manually screened for additional eligible articles. Grey literature was deliberately excluded to maintain a focus on peer-reviewed empirical research and to ensure the quality and reliability of the synthesized evidence.

### Study selection

The study selection process was conducted in strict accordance with the PRISMA 2020 guidelines to ensure methodological rigor, transparency, and reproducibility (Fig. 1). The selection proceeded through a series of clearly defined and sequential steps that allow readers to follow and replicate the process precisely.

Initially, the comprehensive database search identified a total of 988 records. Following this, a systematic deduplication process was undertaken, during which 345 duplicate records were removed, leaving 643 unique citations for screening. These records then underwent title screening to assess relevance to the review objectives, resulting in the exclusion of 295 articles that were clearly unrelated to concept mapping or mind mapping in pediatric nursing education. The remaining 348 records proceeded to abstract screening. At this stage, 283 studies were excluded because they did not meet the predefined eligibility criteria, such as inappropriate population, non-experimental study design, or absence of relevant educational outcomes. Subsequently, 65 articles were identified as potentially eligible and were sought for full-text retrieval. One article could not be accessed, leaving 64 full-text studies that were assessed in detail for eligibility. Following full-text assessment, 52 studies were excluded for specific reasons, including population mismatch, unsuitable study design, lack of measurable outcomes, or insufficient description of the educational intervention. Ultimately, 12 studies met all inclusion criteria and were included in the final review. A detailed breakdown of the number of records excluded at each stage and the reasons for exclusion is presented in Fig. 1 to support transparency and enable replication of the selection process.

Throughout all stages of study selection, two reviewers independently screened the records to minimize selection bias. Any disagreements were resolved through discussion, and when consensus could not be reached, a third reviewer was consulted. This rigorous, multi-step approach ensured that only studies directly aligned with the review objectives were included.

### Data extraction

Data were systematically extracted from each included study using a structured form. The following information was captured: author(s), year of publication, country of origin, population and sample size, study design, details of the concept mapping intervention (such as duration, format, and integration into teaching or training), outcome measures, and key findings.

This standardized approach allowed the findings to be organized clearly, facilitating comparison across studies and enabling the construction of summary tables specific to pediatric nurses and nursing students.

### Quality appraisal

The methodological rigor of the included studies was evaluated using the Joanna Briggs Institute (JBI) critical appraisal checklists, tailored to the respective study designs, including randomized controlled trials and quasi-experimental designs. The appraisal process systematically examined key domains such as the clarity and appropriateness of participant recruitment and selection, the suitability and fidelity of the educational interventions, the comparability of intervention and control groups, and the reliability and validity of the outcome measures employed.

Each study underwent independent evaluation by two reviewers to ensure objectivity and methodological transparency. Discrepancies between reviewers were resolved through consensus discussions, thereby strengthening the consistency of the appraisal process. Studies were subsequently categorized according to the proportion of appraisal criteria met: high quality (≥ 75% of criteria fulfilled), moderate quality (50–74%), or low quality (< 50%). These quality ratings were incorporated into the synthesis to guide interpretation of the strength and reliability of the evidence. This structured approach ensured that conclusions regarding the impact of concept mapping and related strategies on pediatric nursing staff and nursing students were informed by a clear understanding of the methodological robustness of the included studies.

### Data synthesis

Given the heterogeneity across included studies in terms of study design, educational interventions, participant populations, and outcome measures, a meta-analysis was not deemed appropriate. Therefore, a narrative synthesis approach was employed to systematically summarize and integrate the findings in accordance with established guidance for narrative reviews.

The synthesis process involved organizing studies into analytically meaningful groups based on participant type (pediatric nurses working in clinical settings versus nursing students enrolled in pediatric or child health courses). Within each group, studies were further examined according to intervention characteristics, including the type of mapping strategy applied (concept mapping or mind mapping), mode of implementation (individual or collaborative), duration and frequency of the intervention, and the educational context (academic instruction or clinical training).

Outcome measures reported in the included studies were categorized into predefined domains, namely knowledge-related outcomes, critical thinking and clinical reasoning, self-efficacy, problem-solving abilities, and clinical or educational performance indicators. This structured categorization facilitated systematic comparison across studies while accounting for variations in measurement instruments and assessment methods.

The narrative synthesis was conducted in a transparent and reproducible manner, focusing on identifying patterns, similarities, and differences in intervention implementation and outcome assessment, without quantitative pooling of results. This approach ensured that the synthesis accurately reflected the methodological diversity of the included studies while providing a coherent framework for interpreting the evidence in subsequent sections of the review.

## Results

### Impact of mapping-based teaching strategies on pediatric nurses’ learning outcome

Three quasi-experimental studies conducted in Egypt evaluated the impact of concept mapping and mind mapping on pediatric nurses’ knowledge and clinical practice (Table 1). Abdelrahman et al. [5] found significant improvements in pediatric nurses’ knowledge of congenital heart disease (CHD) and training satisfaction after implementing a concept mapping program at Suez Canal University Hospitals [5]. Similarly, Ayed et al. [3] reported that mind mapping enhanced both knowledge and infection control practices among neonate intensive care unit (NICU) nurses at Tanta Main University Hospitals [3].

**Table 1:** Summary of studies on the impact of concept mapping and mind mapping on pediatric nurses’ knowledge and clinical practice ^*^

No	Author (Year)	Study Setting	Study Design	Population / Sample Size	Research Instruments	Key Findings	Conclusion and Implementations
1	Ayed et al. (2022) [3]	NICU, Tanta Main University Hospitals (Egypt)	Quasi-experimental	70 pediatric nurses	Infection Control & Mind Mapping Questionnaire; Observational checklists	Nurses’ knowledge and infection control practice improved significantly post-training	Mind mapping is effective in enhancing infection control training among NICU nurses
2	Hegazy et al. (2024) [23]	NICU, Sohag University Hospital (Egypt)	Quasi-experimental	50 pediatric nurses	Structured questionnaire; Knowledge test; Observational checklist	Nurses’ knowledge and performance in PICC care improved significantly after mind mapping	Mind mapping should be integrated into NICU nurse training for better knowledge and clinical performance
3	Mohamed Abdelrahman et al. (2021) [5]	NICU, PICU, Pediatric Ward, Suez Canal University Hospitals (Egypt)	Quasi-experimental	48 pediatric nurses (pretest), 46 (posttest)	Knowledge questionnaire on concept mapping; Knowledge questionnaire on CHD; Nurses’ satisfaction questionnaire	Significant improvement in knowledge scores and satisfaction after concept-mapping program	Concept mapping is effective in improving pediatric nurses’ knowledge about CHD and increases training satisfaction

^*^ The full names of the abbreviations in this table are listed in the Abbreviations list

In another study, Hegazy et al. [23] demonstrated that mind mapping improved nurses’ knowledge and clinical performance in peripherally inserted central catheter (PICC) care at Sohag University Hospital [23]. Taken together, these studies provide consistent evidence that concept mapping and mind mapping are effective educational tools for pediatric nurses, leading to better knowledge acquisition, enhanced clinical practice, and increased satisfaction with training programs.

### Impact of mapping-based teaching strategies on pediatric nursing students’ learning outcome

Nine studies investigated the effects of concept mapping and mind mapping on nursing students in pediatric education across diverse settings (Table 2), including Egypt, Türkiye, Iran, and Thailand. These studies consistently highlighted improvements in students’ critical thinking, self-efficacy, clinical performance, and learning outcomes [6, 7, 26, 33, 36, 40].

**Table 2:** Studies on the impact of concept mapping and mind mapping on nursing students in pediatric settings^*^

No	Author (Year)	Study Setting	Study Design	Population / Sample Size	Research Instruments	Key Findings	Conclusion and Implementations
1	Aein & Aliakbari (2017) [4]	Pediatric Clinical Nursing Course, Shahrekord Univ. of Medical Sciences (Iran)	Experimental	60 baccalaureate students	California Critical Thinking Skills Test	Significant increase in CT in concept mapping group vs. control	Concept mapping improves CT skills in pediatric nursing courses
2	Aein & Frouzandeh (2012) [33]	Pediatric Nursing Clinical Course, Iran	Semi-experimental	30 students (3 groups)	Nursing process concept map scoring system	Significant improvement in concept map scores from pre to post	Concept maps support knowledge integration and CT
3	Alavi & Okhovat (2024) [6]	Pediatric Departments, Iran	Quasi-experimental	82 undergraduate nursing students	Nursing Caring Self-Efficacy Scale	Higher caring self-efficacy in concept map group	Concept maps enhance caring self-efficacy in pediatric nursing students
4	Apichutboonchock (2022) [26]	Thailand	R&D (4 phases: needs, development, implementation, evaluation)	64 third-year nursing students	Knowledge & nursing diagnosis skill assessment; Open-question guideline	Improved knowledge and diagnostic skills post-intervention	Mind mapping & peer learning strengthen pediatric nursing education
5	Dinç et al. (2024) [8]	Nursing Dept., Western Black Sea Region, Türkiye	Quasi-experimental	140 nursing students (3rd & 4th year; 70 intervention, 70 control)	Self-Efficacy Scale for Medication Administration in Children; Concept Mapping Effectiveness Scale	Higher self-efficacy and effectiveness scores in intervention group	Concept mapping improves medication administration self-efficacy
6	Ebrahim Ammar et al. (2024) [7]	Pediatric Nursing Dept., Menoufia University (Egypt)	Quasi-experimental	230 third-year pediatric nursing students	Characteristics questionnaire; Watson Glaser Critical Thinking; Nursing process evaluation questionnaire	Significant improvement in critical thinking in concept mapping group	Concept mapping enhances critical thinking in pediatric nursing students
7	Khalili et al. (2022) [40]	Iran	Quasi-experimental	103 nursing students (case scenario vs. concept map)	CPR skills checklist	Significantly higher CPR skills in concept mapping group	Concept maps are superior to case scenarios in CPR learning
8	Mahmoud et al. (2021) [36]	Faculty of Nursing, Ain-Shams University (Egypt)	Quasi-experimental	85 third-year pediatric nursing students	Structured Questionnaire; Concept Map Rubric; Achievement Test; Opinionaire	Higher achievement and satisfaction in concept mapping group	Concept maps enhance student achievement and satisfaction
9	Ramazani et al. (2017) [34]	Iran	Quasi-experimental	64 fifth-semester nursing students	Academic achievement test	Concept mapping produced the greatest gains in meaningful learning	Concept mapping should be prioritized for long-term learning

^*^ The full names of the abbreviations in this table are listed in the Abbreviations list

For example, Ammar et al. [7] reported that concept mapping significantly enhanced critical thinking among 230 third-year pediatric nursing students in Egypt [7]. Similarly, Dinç et al. [8] demonstrated improved self-efficacy in medication administration among nursing students in Türkiye [8], while Aein and Aliakbari [4] found higher critical thinking scores among Iranian students exposed to concept mapping [4]. Alavi and Okhovat [6] confirmed that caring self-efficacy was significantly higher in concept mapping groups [6], and Ramazani et al. [34] emphasized its role in fostering long-term meaningful learning [34].

Further evidence from Mahmoud et al. [36] showed higher academic achievement and satisfaction with concept mapping among pediatric nursing students in Egypt [36]. Likewise, Khalili et al. [40] demonstrated that concept mapping led to better cardiopulmonary resuscitation (CPR) skill acquisition compared to case scenarios [40]. Earlier work by Aein and Frouzandeh [33] revealed sustained improvements in integrating knowledge and critical thinking [33]. Finally, Apichutboonchock [26] in Thailand highlighted that combining mind mapping with peer learning enhanced knowledge acquisition and diagnostic skills [26].

Collectively, these studies affirm that concept mapping and mind mapping are effective educational strategies for pediatric nursing students, leading to enhanced cognitive skills, improved clinical competencies, and higher student satisfaction.

## Discussion

This study is significant because it is the first systematic review to comprehensively evaluate the effects of concept mapping and mind mapping specifically in pediatric nursing education, targeting both nursing staff in clinical pediatric settings and nursing students enrolled in pediatric courses. By synthesizing the available evidence, the review highlights the effectiveness of these educational strategies in bridging the theory–practice gap, fostering critical thinking, and enhancing clinical performance in pediatric care.

The findings of this review demonstrate that concept mapping and mind mapping are effective educational strategies that enhance pediatric nurses’ knowledge and clinical practice, as well as improve learning outcomes among nursing students. Studies consistently reported significant gains in knowledge acquisition, critical thinking, clinical reasoning, and self-efficacy, reflecting the potential of these approaches to address gaps in pediatric nursing education and practice. For instance, Abdelrahman et al. [5] found that concept mapping programs significantly improved nurses’ knowledge of congenital heart disease [5], while Ayed et al. [3] and Hegazy et al. [23] reported improved infection control and PICC care performance among neonatal nurses following mind mapping interventions [3, 23]. These outcomes highlight the direct contributions of structured educational strategies to safer, evidence-based pediatric nursing care.

Among nursing students, integrating concept mapping into pediatric education was associated with improvements across multiple domains. Aein and Aliakbari [4] and Ammar et al. [7] reported significant increases in critical thinking skills [4, 7], while Dinç et al. [8] observed enhanced self-efficacy in pediatric medication administration [8]. Mahmoud et al. [36] and Ramazani et al. [34] found that students exposed to concept mapping achieved higher academic outcomes compared to peers taught through traditional lecture-based approaches [34, 36]. Similarly, Khalili et al. [40] demonstrated superior CPR performance among students taught using concept maps compared with case scenario teaching, underscoring the value of this method in promoting psychomotor skills essential for pediatric care [40]. Collectively, these findings align with broader meta-analyses that confirm the effectiveness of concept mapping in nursing education for improving critical thinking and knowledge retention [1, 2].

The broader implications extend beyond cognitive outcomes to professional competence and patient safety. Enhanced infection control practices [3], improved clinical decision-making in PICC care [23], and better understanding of pediatric pathophysiology [5] can directly reduce complications and support recovery in critically ill children. Similarly, the development of self-efficacy and caring confidence among students [6, 44] suggests that concept mapping fosters holistic care and prepares graduates for complex pediatric environments. These results resonate with the wider nursing literature, which shows that concept mapping strengthens clinical judgment, promotes reflective practice, and supports evidence-based nursing care [14, 15, 35].

This review also identifies important gaps in the existing evidence. Although quasi-experimental studies dominate the literature, few randomized controlled trials have been conducted among pediatric nurses, limiting the strength of conclusions. Moreover, most studies measured immediate learning outcomes without assessing long-term knowledge retention or clinical impact. The lack of standardized outcome measures, especially regarding critical thinking and self-efficacy, further complicates comparisons across studies [46, 47]. Additionally, digital and hybrid approaches to concept mapping remain underexplored, despite promising evidence of their feasibility and effectiveness in other educational contexts [27, 43].

### Study implications and recommendations

This review demonstrates that concept mapping and mind mapping are effective pedagogical strategies for advancing pediatric nursing education in both clinical and academic contexts. Across the included studies, these approaches consistently improved knowledge acquisition, critical thinking, clinical reasoning, self-efficacy, and practical skills among nursing students and pediatric nursing staff when compared with traditional teaching methods [2, 4–6]. For nursing staff, particularly those working in neonatal and pediatric intensive care units, mapping strategies enhanced professional competence and knowledge retention in specialized areas such as infection control, peripherally inserted central catheter care, and management of congenital heart diseases [48–51]. These improvements directly contribute to safer and higher-quality pediatric care, underscoring the value of integrating mapping into in-service education and professional development programs to strengthen evidence-based practice and clinical decision-making [3, 5, 23]. For nursing students, concept mapping supports deeper understanding of pediatric care processes, fosters academic achievement, and enhances decision-making in complex scenarios, while mind mapping encourages interactive learning and diagnostic reasoning [15, 26, 36]. Embedding mapping strategies into pediatric curricula, simulation exercises, and clinical skill laboratories, together with constructive faculty feedback and collaborative learning opportunities, can maximize educational impact [6–8, 14].

Despite the promising outcomes, further research is needed to strengthen the evidence base. Future investigations should employ multi-center randomized controlled trials across diverse pediatric nursing settings to enhance generalizability [1, 2], examine the long-term impact of mapping strategies on clinical practice, patient safety, and family-centered care, and evaluate digital and hybrid approaches such as mobile applications, e-learning, and simulation-based mapping to identify the most effective delivery methods [43, 44]. Qualitative studies exploring barriers, facilitators, and learners’ experiences would also provide important insights for curriculum design and practical implementation in real-world pediatric care [45].

In conclusion, mapping-based teaching strategies represent evidence-based, adaptable, and transformative strategies that can enhance the competence of pediatric nursing students and staff alike. By fostering critical thinking, clinical reasoning, and self-efficacy, these approaches bridge the gap between theoretical knowledge and clinical application, ultimately improving the quality of pediatric care and health outcomes for children and their families [5, 7]. Integrating these strategies into curricula, professional training, and future research agendas offers a pathway toward more competent, confident, and patient-centered pediatric nursing care.

### Study strengths and limitations

This systematic review has several strengths. It synthesizes evidence from studies conducted across multiple countries and includes both pediatric nurses and nursing students, providing a comprehensive understanding of concept mapping’s impact on professional practice, academic learning, and cognitive development. The included studies used quasi-experimental and experimental designs with pretest–posttest comparisons, which enhances the reliability of the findings. Additionally, validated instruments such as critical thinking tests, self-efficacy scales, clinical performance checklists, and structured knowledge questionnaires were employed, supporting the accuracy of measured outcomes. Overall, the review demonstrates consistent positive effects of mapping-based teaching strategies on knowledge, critical thinking, clinical performance, and learning satisfaction, highlighting their practical value in pediatric nursing education and practice.

However, some limitations should be noted. Most studies used quasi-experimental designs without randomization, which may limit causal interpretations, and sample sizes were often small, reducing generalizability. The interventions, outcomes, and educational contexts varied across studies, making direct comparisons and synthesis challenging. Furthermore, few studies assessed long-term knowledge retention or the sustained impact on patient care, restricting conclusions about enduring benefits. Future research should focus on larger, randomized trials with standardized interventions and long-term follow-up to better evaluate the effectiveness of concept mapping in improving pediatric nursing education and patient care outcomes.

## Conclusion

This systematic review provides evidence that concept mapping and mind mapping are effective educational strategies for improving learning outcomes among pediatric nurses and nursing students. Across diverse educational and clinical settings, these approaches were associated with enhanced knowledge organization, critical thinking, self-efficacy, and clinical performance. By supporting the integration and visualization of complex pediatric nursing concepts, mapping-based strategies facilitate deeper understanding and more effective clinical reasoning in both academic and practice-based contexts.

The findings support the integration of concept mapping and mind mapping into pediatric nursing curricula and continuing professional education as structured, learner-centered teaching strategies. However, further research using rigorous experimental designs, standardized assessment tools, and longitudinal follow-up is needed to confirm the durability of these effects and to better understand how mapping-based educational interventions influence clinical practice in pediatric nursing. 

## Data Availability

No datasets were generated or analysed during the current study.

## Abbreviations

NICU: Neonatal Intensive Care Unit
PICU: Pediatric Intensive Care Unit
PICC: Peripherally Inserted Central Catheter
CHD: Congenital Heart Disease
CT: Critical Thinking
CCTST: California Critical Thinking Skills Test
R&D: Research and Development
CPR: Cardiopulmonary Resuscitation
WGCTA: Watson–Glaser Critical Thinking Appraisal
